# Late-Onset Periodic Fever, Aphthous Stomatitis, Pharyngitis and Cervical Adenitis (PFAPA) Syndrome: A Cue for Antimicrobial Stewardship

**DOI:** 10.7759/cureus.99478

**Published:** 2025-12-17

**Authors:** Kalyani Parvathy, Aravind Reghukumar, Athul Gurudas, Kirankumar V S, Parvathy J

**Affiliations:** 1 Respiratory Medicine, Ealing Hospital, Southall, GBR; 2 Internal Medicine, Government Medical College, Thiruvananthapuram, Thiruvananthapuram, IND; 3 Infectious Diseases, Government Medical College, Thiruvananthapuram, Thiruvananthapuram, IND; 4 Infectious Diseases, Government Medical College, Kottayam, Kottayam, IND; 5 Microbiology, Government Medical College, Thiruvananthapuram, Thiruvananthapuram, IND

**Keywords:** acute pharyngitis, antimicrobial stewardship, autoinflammatory disorder, periodic fever syndrome, pfapa

## Abstract

PFAPA is an auto-inflammatory syndrome, characterised by periodic fever, aphthous stomatitis, pharyngitis and cervical adenitis, most commonly described in children. We report two cases of late-onset PFAPA syndrome: a 24-year-old woman and a 28-year-old man, presenting to our tertiary care center in India. In both cases, the diagnosis of PFAPA was made clinically and the patients responded well to treatment with corticosteroids. Late-onset PFAPA syndrome is an emerging but under-recognised clinical entity. Its diagnosis in adults is challenging due to overlap with conditions causing recurrent fever like chronic infections as well as a general lack of awareness of its occurrence in adults. The natural history and long-term outcomes of PFAPA in adults are not yet well-established. Recognising PFAPA or similar auto-inflammatory syndromes in the list of differentials for patients presenting with recurrent fever will help to avoid the misuse of antimicrobial therapies and facilitate targeted management.

## Introduction

Autoinflammatory disorders (AIDs) are one of the common causes of periodic fever syndromes, which, if undiagnosed, are usually treated with antibiotics. The syndrome of periodic fever, aphthous stomatitis, pharyngitis and cervical adenitis is termed ‘PFAPA’ syndrome, and it is the most common periodic fever syndrome in childhood, which rarely might persist to adulthood. It is characterised by episodes of fever lasting for three to six days with recurrence every two to six weeks, associated with at least one of the three main symptoms, that is, aphthous stomatitis, pharyngitis, or cervical lymphadenitis [1]. PFAPA syndrome, also known as Marshall’s syndrome, is an immune-mediated disease characterised by cytokine dysfunction. Patients with PFAPA syndrome respond to treatment with immunomodulators like corticosteroids, colchicine, etc., and antibiotics have no role in the management of this condition [2]. The incidence or prevalence of PFAPA in adults has not been defined. Due to the rare occurrence of adults presenting with PFAPA, there are significant delays in diagnosing PFAPA in adults when compared with children, and the long-term outcome of PFAPA in adults is not established [3]. We report two consecutive cases of PFAPA syndrome diagnosed in adulthood, which we managed in our tertiary care center and are on follow-up.

## Case presentation

Case 1

**Figure 1:**
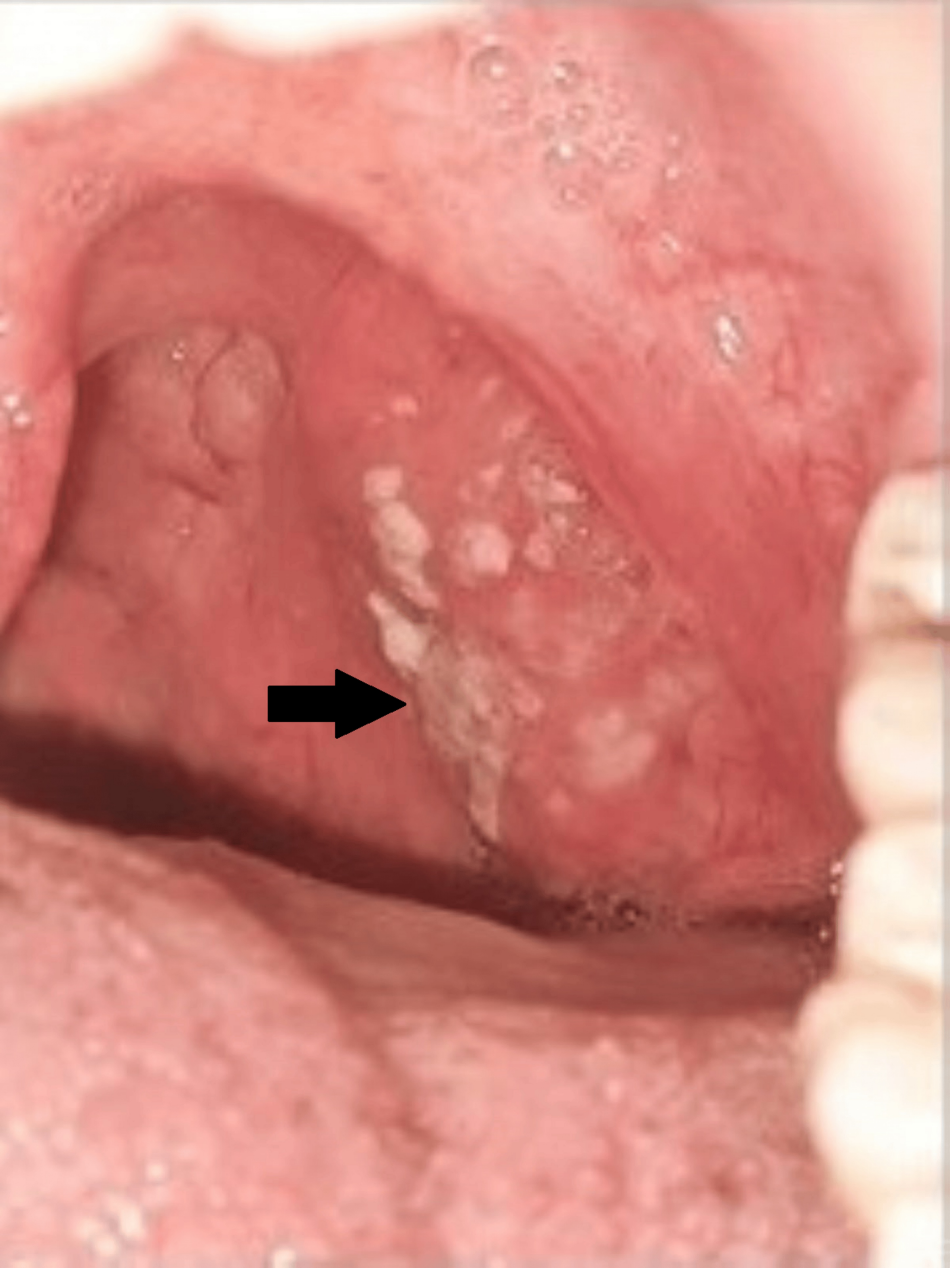
Tonsillar ulcers in the oral cavity Arrow pointing to shallow ulcer seen at tonsillar pillar

In 2017, a 24-year-old woman was referred in view of recurrent episodes of fever, erythematous pharyngitis, aphthous ulcers and cervical lymphadenitis. Each episode used to last for 4 to 5 days, and she was prescribed oral antibiotics for the symptoms. She had recurrent episodes every three weeks for the previous two years. During the period between each episode, she was asymptomatic and active. There were no other features suggestive of vasculitis (including features of Behcet's like uveitis or genital ulcers). No other features of connective tissue disease were present and no history suggestive of rheumatic fever in the past. The episodes were not related to the patient’s menstrual cycle and there was no family history of similar complaints. Physical examination was suggestive of tonsillar ulcers in the oral cavity and enlarged cervical lymph nodes (Figure 1).

Laboratory workup revealed raised ESR and CRP during every episode without neutropenia. Throat swab culture was sterile in all episodes. ASO titers were normal and workup for vasculitis was negative (Table 1). Serologies for HIV, EBV and CMV were negative. Fine needle aspiration cytology (FNAC) of the cervical lymph node showed reactive changes. In view of the recurrent episodes, the patient was started on oral penicillin prophylaxis but it failed to prevent the episodes from occurring. Recurrent febrile episodes with strict periodicity and failure to respond to antibiotic prophylaxis pointed towards a possibility of a periodic fever syndrome. Monogenic syndromes like familial Mediterranean fever (FMF) were ruled out as there was no family history; however, genetic testing was not performed. The patient’s clinical features and laboratory findings were found to meet the diagnostic criteria of PFAPA; hence, prednisone was prescribed for acute treatment of the attacks. The patient showed dramatic improvement with the therapy and is being kept on follow-up. Since then, she has had reduced frequency of episodes, ranging from 2 to 3 per year, and takes a short course of steroids during the attacks.

**Table 1:** Case 1 - Blood investigation reports SGOT: Serum glutamic-oxaloacetic transaminase; SGPT: serum glutamate pyruvate transaminase; ALP: alkaline phosphatase

Lab parameter	Value	Range	Unit
Hb	13.5	12-16	g/dL
Total leukocyte count	11000	4000-11000	cells/mm^3^
Neutrophils	60	40-60	%
Lymphocytes	38	20-40	%
Platelets	2.5	1.5-4.5	lakhs/mm^3^
Creatinine	0.8	0.8-1.3	mg/dL
Total bilirubin	1.2	0.2-1.2	mg/dL
SGOT	28	upto 35	U/L
SGPT	26	upto 36	U/L
ALP	140	64-306	U/L
CRP	24	<10	mg/L
ESR	50	<20	mm/h
ASO titre	180	<200	IU/ml
ANA	Negative		
anti-ds DNA	Negative		
RA factor	Negative		

Case 2

**Figure 2:**
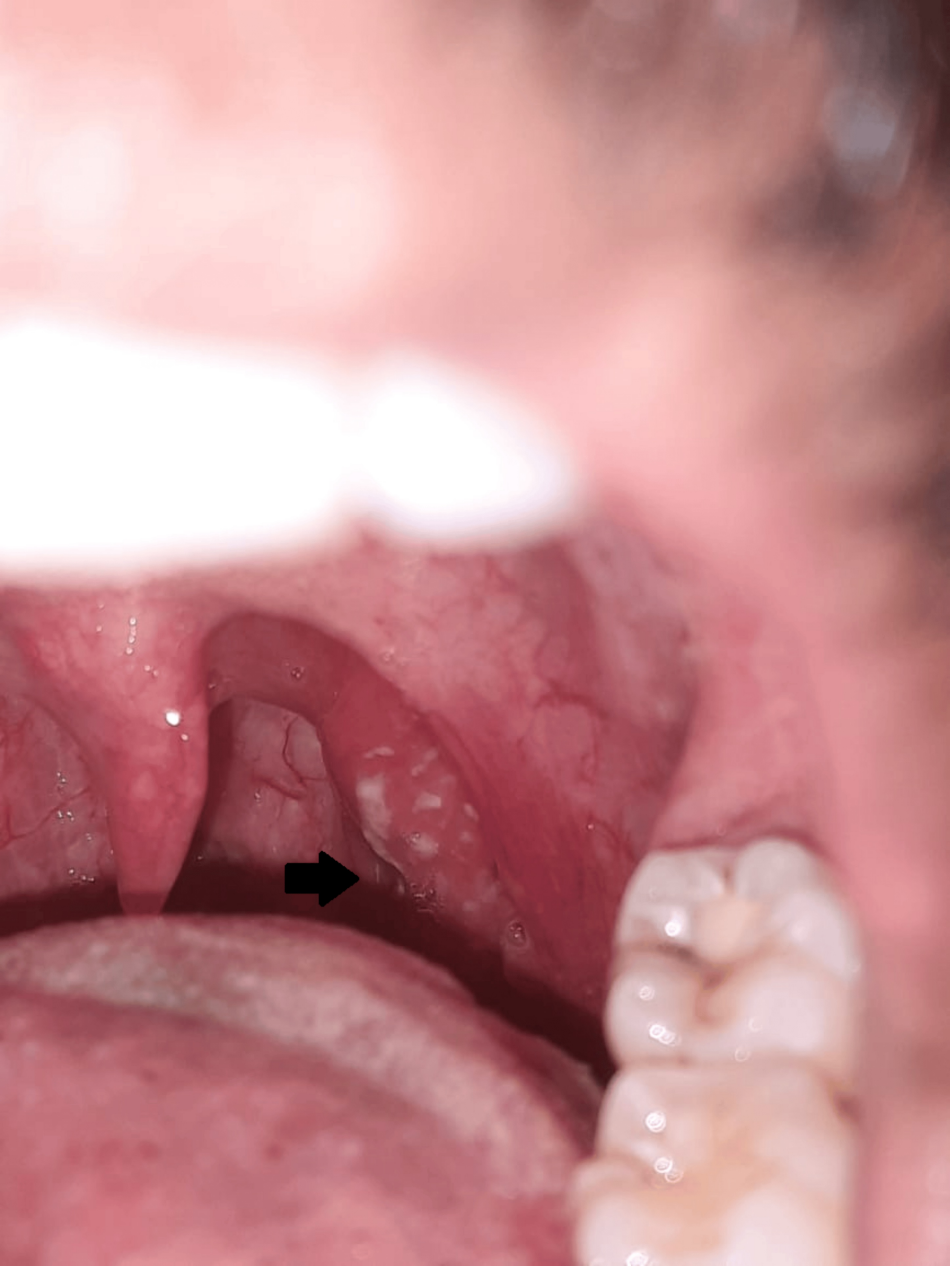
Exudative pharyngitis Arrow pointing to pus spots over tonsils

In 2022, a 28-year-old man with a history of recurrent episodes of fever, exudative pharyngitis, aphthous ulcers and cervical lymphadenitis from the age of nine years was referred to the Infectious Diseases department from an ENT specialist for evaluation. Episodes used to recur every three weeks and used to last for 3 to 5 days. The patient had been prescribed antibiotics during each episode since the onset of symptoms in childhood. His growth and development in childhood were normal. He had no history suggestive of vasculitis or connective tissue diseases (Figure 2). 

Inflammatory markers (CRP and ESR) were elevated during each episode and there was no neutropenia. Vasculitis workup and ASO were negative (Table 2). Serologies for HIV, EBV and CMV were negative. Throat swab was sterile. FNAC of the cervical lymph node showed reactive changes. As the clinical profile was suggestive of PFAPA syndrome, he was initiated on corticosteroids (methylprednisolone 8 mg twice daily) for three days with which he had dramatic improvement, which was observed on the first day of initiation. The patient experienced a recurrent episode after three weeks which responded to a single dose of corticosteroid. He was initiated on colchicine prophylaxis to reduce the frequency of flares and is on follow-up. He has had no further episodes since initiation of colchicine prophylaxis.

**Table 2:** Case 2 - Blood investigation reports SGOT: Serum glutamic-oxaloacetic transaminase; SGPT: serum glutamate pyruvate transaminase; ALP: alkaline phosphatase

Lab parameter	Value	Range	Unit
Hb	14.2	14-18	g/dL
Total leukocyte count	7000	4000-11000	cells/mm^3^
Neutrophils	64	40-60	%
Lymphocytes	36	20-40	%
Platelets	3.4	1.5-4.5	lakhs/mm^3^
Creatinine	0.9	0.8-1.3	mg/dL
Total bilirubin	0.2	0.2-1.2	mg/dL
SGOT	12	upto 35	U/L
SGPT	14	upto 36	U/L
ALP	104	64-306	U/L
CRP	28	<10	mg/L
ESR	56	<20	mm/h
ASO titre	150	<200	IU/ml
ANA	Negative		
anti-ds DNA	Negative		
RA factor	Negative		

## Discussion

The earliest mention of a disease with a periodic nature was in 1802, when Heberden described a patient with recurrent episodes of abdominal pain and painful extremities [1]. Periodic fevers were reported over a century later by Reimann and deBarardini. It is now believed that periodic fevers fall under a broader spectrum of autoinflammatory syndromes, which are characterised by recurrent fever along with involvement of the eyes, skin, joints, and muscles. Autoinflammatory syndromes differ from autoimmune diseases in that there are no autoantibodies or autoantigens involved in the former; rather, they are a result of immune dysregulation, specifically of innate immunity. A majority of the autoinflammatory disorders are inherited, with certain ethnic predilections and childhood onset [1]. PFAPA syndrome is one such autoinflammatory syndrome.

The onset of PFAPA syndrome is usually before the age of five years, and the fever episodes subside within five years of onset or in the pre-pubertal years [3]. Though rare, PFAPA has been reported in adults as well. Padeh et al. reported PFAPA in 15 adults, of whom two patients had their first attack in childhood [4]. Both the patients in our case report had a late onset of PFAPA; while one patient’s symptoms started in late childhood and continued into adulthood, the other patient presented in adulthood. It is still unclear whether adult-onset PFAPA is a separate entity or merely the continuation of symptoms unnoticed from childhood.

The pathogenesis of PFAPA is yet to be fully understood. It is now considered to be a complex genetic disorder, with some of the genetic risk loci overlapping with Behcet’s syndrome and recurrent aphthous ulcers. The risk variants associated with PFAPA suggest that there could be elevated activity of CD4+ Th1 and Th17 lymphocytes during flares of the disease, along with decreased IL-10 (which is an anti-inflammatory cytokine) [5]. It is speculated that there could be environmental triggers inducing syndrome onset in individuals with a genetic risk for PFAPA. One hypothesis suggested for the development of PFAPA in adults is that the immune system in affected adults had not attained complete maturation, making them prone to stimuli that trigger episodes of PFAPA [4].

PFAPA is characterised by brief febrile episodes that last around five days, which are associated with chills, headache, and myalgia. It is accompanied by one of the “APA” symptoms, namely (i) Aphthous stomatitis - painful aphthous ulcers can be observed on the lips and buccal mucosa, which heal without scarring, (ii) Pharyngitis - erythematous, with or without exudates, (iii) Adenopathy - specifically cervical lymphadenitis. These episodes of fever recur every two to six weeks, with the patient being symptom-free between the episodes and showing normal growth and development [1].

The clinical presentation of PFAPA in adults is largely similar to the syndrome in children; however, it has been reported that constitutional symptoms like fatigue, malaise, arthralgia, myalgia, and headaches are more common in adults. The fever episodes also do not always occur with strict periodicity like those seen in children, and exudative pharyngitis is not seen commonly in adults with PFAPA [3].

Differentials that are important to be considered with these clinical findings include infections, other autoinflammatory diseases, and rare inherited diseases like cyclic neutropenia. Features that point away from an infectious etiology include the symptom-free intervals with definite regularity in PFAPA, absence of geographic clustering, and lack of further cases among close contacts. Other features that suggest a non-infectious cause include the failure to respond to antibiotics and the response to corticosteroids [4]. Cyclic neutropenia presents with similar symptoms of fever and mouth ulcers and can be distinguished by identifying the exact pattern of fever by keeping a fever diary, monitoring blood counts for neutropenia, and by genetic testing for mutations in the ELANE gene. PFAPA shows a clinical overlap with inherited autoinflammatory disorders like FMF. While PFAPA is a polygenic disease, monogenic autoinflammatory diseases like FMF are often distinguishable as they will show an existing hereditary pattern. Laboratory findings during these episodes include a transient leucocytosis and a modest elevation of ESR and CRP values. However, diagnosis of PFAPA is made on clinical grounds after exclusion of other causes, and no specific diagnostic tests are available to date [1]. We acknowledge that a limitation in both our cases was that we had not conducted genetic testing for Familial Mediterranean Fever and ruled it out based on the lack of family history.

PFAPA was first described by Marshall et al. in 1987 using a set of clinical criteria, which was confirmed by Thomas et al. in 1999 [6]. Criteria include: (1) Recurrent febrile episodes beginning in early childhood (before five years of age), (2) Systemic symptoms occurring without evidence of an upper respiratory tract infection, accompanied by at least one of the following: aphthous ulcers (stomatitis), pharyngitis and/or cervical lymph node enlargement (lymphadenitis), (3) Ruling out cyclic neutropenia, (4) Complete return to baseline health between attacks, and (5) Normal growth and developmental progress.

Cantarini et al. in 2017 proposed a set of diagnostic criteria for adult-onset PFAPA (refer to Table 3). Notably, it does not include age, aphthous stomatitis, neutropenia or normal growth as criteria and has included increased inflammatory markers during attacks as a diagnostic criterion [7].

**Table 3:** Diagnostic criteria for adult-onset PFAPA syndrome Reproduced from [7] under Creative Commons Attribution License (CC BY) PFAPA: Periodic fever, aphthous stomatitis, pharyngitis and cervical adenitis

SI No	Diagnostic criteria for adult-onset PFAPA syndrome
1	Recurrent fever accompanied by:
	a) Erythematous pharyngitis and/or
	b) Cervical lymphadenitis
2	Increased inflammatory markers during attacks
3	Symptom-free intervals

While both the patients in our report have a clinical picture fitting into the diagnostic criteria proposed by Cantarini et al. [7], the second patient, whose symptoms started in late childhood and persisted into adulthood, represents a possible third group of patients who do not fully belong to either set of diagnostic criteria mentioned in this report due to the age of onset of symptoms.

Medical treatment is effective during PFAPA flares, but there is no evidence that it modifies the outcome. Oral glucocorticoids are the mainstay of treating a flare. The use of anti-pyretics and non-steroidal anti-inflammatory agents has not been found to be fully effective in treating a febrile episode. Response to corticosteroids is also helpful to distinguish between attacks of PFAPA and FMF. However, corticosteroids have not been found to prevent further attacks of PFAPA. There is limited data available on prophylactic therapy for PFAPA, and some studies have reported colchicine to have some effect in prevention but not necessarily induce complete remission [2]. Another medication that has been tried in the management of PFAPA includes cimetidine, an H2 antagonist that has immunomodulating properties. A randomised controlled trial done in 67 children with PFAPA showed that treatment regimens with cimetidine are as effective as colchicine in preventing febrile attacks [8]. Tonsillectomy has been shown to have a beneficial effect on the disease course in children and can be considered when the interval between attacks is short as it makes the use of corticosteroids inappropriate [2].

PFAPA has a favourable natural history and is self-limiting. Most children experience spontaneous resolution of fever episodes by adolescence. A few patients may get persistent symptoms in adulthood; however, these episodes are not as frequent and are for a shorter duration [9]. The long-term outcome of PFAPA presenting in adults is not known, and the occurrence of spontaneous resolution in them remains a mystery.

## Conclusions

PFAPA is a syndrome commonly described in children; however, symptoms can start later in childhood or in adulthood. The febrile episodes can mimic upper respiratory infections and should be differentiated by the periodicity of symptoms and symptom-free intervals to avoid inappropriate administration of antibiotics. Corticosteroids are used to treat acute attacks, and colchicine may have some benefit as prophylaxis. PFAPA is self-limiting in children, but the course of the disease in adults is yet to be understood. Timely diagnosis of AIDs is essential for antimicrobial stewardship. Multifactorial AIDs like PFAPA syndrome usually resolve by adolescence, but there are an increasing number of cases persisting up to adulthood getting reported. Clinicians should be aware of these conditions to avoid misuse of antimicrobials.
